# Strong relations of elbow excursion and grip strength with post-stroke arm function and activities: Should we aim for this in technology-supported training?

**DOI:** 10.1177/2055668318779301

**Published:** 2018-08-12

**Authors:** Sharon M Nijenhuis, Gerdienke B Prange-Lasonder, Judith FM Fleuren, Jan Wagenaar, Jaap H Buurke, Johan S Rietman

**Affiliations:** 1 Roessingh Research and Development and Roessingh Rehabilitation Centre, Enschede, the Netherlands; 2 Department of Biomechanical Engineering, University of Twente, Enschede, the Netherlands; 3 Department of rehabilitation medicine, ZGT Hospital, Almelo, the Netherlands; 4 Physical Therapy and Human Movement Sciences, Northwestern University, Chicago, IL, USA; 5 Department of Biomedical Signals and Systems, University of Twente, Enschede, the Netherlands

**Keywords:** Stroke, upper extremity, kinematics, clinical outcome measures, recovery of function, reaching and grasping

## Abstract

**Objective:**

To investigate the relationships between an extensive set of objective movement execution kinematics of the upper extremity and clinical outcome measures in chronic stroke patients: at baseline and after technology-supported training at home.

**Methods:**

Twenty mildly to severely affected chronic stroke patients participated in the baseline evaluation, 15 were re-evaluated after six weeks of intensive technology-supported or conventional arm/hand training at home. Grip strength, 3D motion analysis of a reach and grasp task, and clinical scales (Fugl-Meyer assessment (FM), Action Research Arm Test (ARAT) and Motor Activity Log (MAL)) were assessed pre- and post-training.

**Results:**

Most movement execution parameters showed moderate-to-strong relationships with FM and ARAT, and to a smaller degree with MAL. Elbow excursion explained the largest amount of variance in FM and ARAT, together with grip strength. The only strong association after training was found between changes in ARAT and improvements in hand opening (conventional) or grip strength (technology-supported).

**Conclusions:**

Elbow excursion and grip strength showed strongest association with post-stroke arm function and activities. Improved functional ability after training at home was associated with increased hand function. Addressing both reaching and hand function are indicated as valuable targets for (technological) treatment applications to stimulate functional improvements after stroke.

## Introduction

Upper extremity hemiparesis is a major problem in patients with stroke, affecting their independence in performance of daily life activities.^1^ Therefore, optimal recovery of arm and hand function is an important goal in stroke rehabilitation. Essential treatment aspects for neurorehabilitation are intensive practice with active engagement of the patient, performing meaningful task-specific exercises in a high dose.^2–4^ Technology-supported treatment can facilitate independent, self-administered training with many repetitions and enhance the dosage of treatment, especially when applied in a (partly) therapist-independent setting, for instance, at home. Although several studies have shown that technology-supported interventions are effective to improve upper extremity motor function after stroke, their influence on activity level is less understood.^5–7^ This might be explained by the fact that many of those technology-supported interventions are focused on body function level, even though ultimately its impact is desired on activity level.^6^

Another factor which might contribute to the limited understanding of how technology-supported interventions can influence performance on activity level is an inappropriate choice of outcome measures.^8–10^ Inclusion of outcome measures covering all domains of the International Classification of Functioning, Disability and Health (ICF) is recommended.^11^ However, when clinical outcome measures are applied to quantify those domains, improvements in, for instance, activity level cannot be attributed to either recovery or compensation. “Recovery” is used in this paper to describe improvements resulting from restitution or repair of structures and functions, “compensation” is defined as the appearance of alternative movement patterns or the use of alternate joints or end effectors during the accomplishment of a task.^12^ In order to differentiate between recovery and compensation, more detailed information of movement patterns and strategies is needed, collected in an objective and reliable way.^12–14^ This is usually not part of standardized clinical outcome measures since they mainly focus on task accomplishment. However, kinematic movement analysis can provide valuable information on the quality of functional task performance, at least when assessed in a research setting.

With a better understanding of the relation between objective movement execution parameters of the affected arm and hand after stroke (as assessed via kinematics and grip strength) and sensorimotor function or activity limitations (as assessed via clinical outcome measures), we gain more insight into underlying mechanisms and may be able to specify areas of attention for (design of) upper extremity interventions.^12,15,16^ Moreover, the effect of an intervention on restoration of function by recovery or compensation might be distinguished, in order to better understand how the intervention affects the restoration capacity of a patient.

Previous research in stroke patients showed significant relations between kinematic outcomes measured during a reach and grasp task and sensorimotor function and activity limitation of the upper extremity.^17,18^ Movement smoothness (MS) and total movement time (MT), together with compensatory trunk displacement were associated with activity capacity (assessed by the Action Research Arm Test (ARAT)).^17^ Another study found trunk displacement alone explaining the majority of variance in sensorimotor function (assessed by the Fugl-Meyer assessment (FM)).^18^ Kinematic variables assessed during a reaching task (including endpoint variables, trunk involvement, joint recruitment, and interjoint coordination) were significant predictors for improvement in self-perceived activity performance in daily life, as measured with the Motor Activity Log (MAL) in mild to moderate chronic stroke.^19^ These studies show partly overlapping results on one hand, but on the other hand tend to differ, with various kinematic outcome measures used in different tasks. A study with a comprehensive set of movement execution parameters is desired in order to provide a more complete picture.

Therefore, we determined the relationships between an extensive set of movement execution parameters (measured via kinematics) during a functional reach and grasp task and grip strength and outcomes on sensorimotor function, activity capacity, and self-perceived activity performance (measured via clinical outcome measures) in mildly to severely affected chronic stroke patients. To obtain a more in-depth insight into the role of recovery versus compensation, we examined whether and how training-induced changes in movement execution parameters were related to training-induced changes in clinical outcome measures after technology-supported or conventional arm and hand training at home.

## Methods

### Participants

Kinematic data obtained during two previous studies on chronic stroke patients within the Supervised Care and Rehabilitation Involving Personal Telerobotics (SCRIPT) project^20^ were combined in the current work for additional analysis: a cross-sectional measurement in which direct effects of a passive dynamic wrist and hand orthosis on hand and arm movement kinematics were assessed^21^ and a randomized controlled trial (RCT) with six weeks of intensive, self-administered arm and hand training at home.^22^ All participants signed informed consent forms before inclusion into either study, approved by the medical ethical committee Twente, Enschede, the Netherlands and registered at the Netherlands Trial Registry (NTR3669). Both studies had the same inclusion criteria: > 6 months post-stroke, age between 18 and 80, movement limitations in the arm and/or hand, but with at least 15° active elbow flexion and able to actively flex the finger(s) by at least 25% of the passive range of motion, live at home with internet access, and able to understand and follow instructions. Exclusion criteria were orthopedic or neurological disease and/or pain restricting active range of motion of the upper extremity.

### Procedures

During a cross-sectional measurement (pre), 20 participants underwent clinical measurements to evaluate status of arm and hand function and dexterity, hand grip dynamometry to measure maximal grip strength and a functional reach and grasp task to determine movement execution. All outcome measures were evaluated at the affected body side. A subset of 15 participants repeated the same measurements after six weeks of intensive training for the arm and hand at home (post). These participants used either a technology-supported training system (experimental group) or performed conventional exercises from an exercise book (control group). This exercise book contained several arm and hand exercises with varying complexity. The technology-supported training system consisted of a passive orthosis providing extension forces to the wrist and fingers to support wrist extension and hand opening (HO), an arm support device, and computer containing gaming exercises for training of the arm and hand. The details of both interventions are described elsewhere.^22^

#### Clinical outcome measures

The sensorimotor function of the arm was measured with the upper extremity part of the FM. The FM assesses the ability to perform isolated movements of the arm, wrist, hand, and coordination within and out of synergy. The maximal score is 66.^23,24^ Activity capacity was evaluated by the ARAT. The ARAT evaluates dexterity on the subtests grasp, grip, pinch, and gross arm movements, with a maximal score of 57.^25,26^ The MAL was used to assess self-perceived activity performance, in terms of amount of use (AOU) and quality of movement (QOM) of the paretic arm and hand during activities of daily life. The MAL is a semi-structured interview with 26 items and has a maximal score of 5 for both subsections.^27^

#### Grip strength

The maximal grip strength of the affected hand was measured using a hand-held dynamometer, while the participants sat on a chair, with the shoulder adducted, the elbow flexed 90° and neutral position of the forearm and wrist. Participants were verbally encouraged to squeeze the dynamometer with maximal strength. The best result from three repetitions, separated by 15 s of rest, was used for analysis.^28^

#### Reach and grasp task

**Figure 1.**
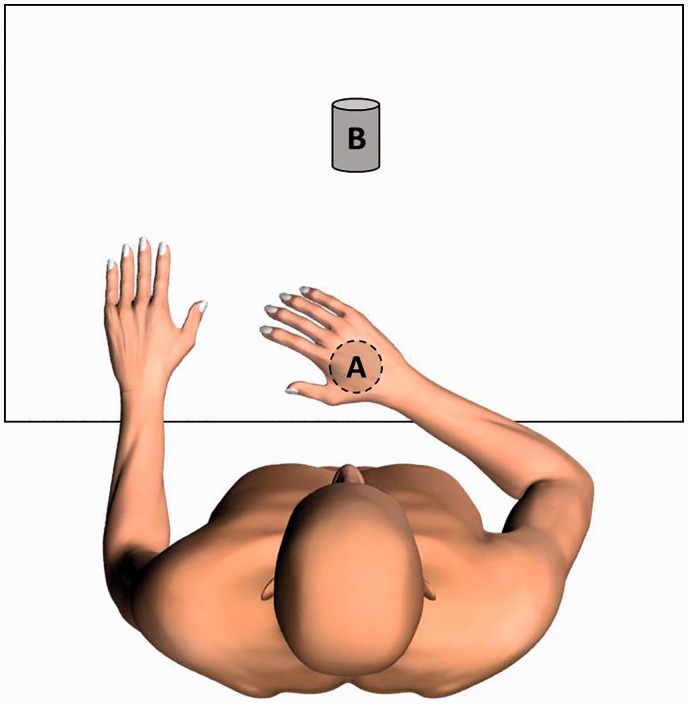
Measurement setup during reach and grasp task.

A standardized reach and grasp task was performed to measure upper extremity movement execution during a functional task, related to activities of daily living. Each participant sat on a chair, with the shoulder adducted, the elbow in 90° flexion, with the palm of the hand resting on the table in front of the participant (Point A in Figure 1). The reach and grasp task involved four phases: forward reaching to a bottle with diameter of 6 cm placed on the table and grasping it (Point B in Figure 1), holding the bottle while moving the arm to the start position (Point A), bringing it back to the original position (Point B) on the table and releasing the bottle, and returning the hand to the start position. The distance of the start position to the bottle was determined by near-maximal (approximately 80%) active forward reach at the start of the task. The participant was instructed to perform the reach and grasp task with the affected arm and hand, at a comfortable, self-selected speed for about 10 repetitions. The trunk was not constrained during the reach and grasp task, and compensatory trunk movements were allowed and measured.

### Kinematic data analysis

Upper extremity movement kinematics were recorded during the reach and grasp task using a 3D motion analysis system (VICON MX13 + motion capture system, Oxford Metrics, Oxford, UK). Six infrared cameras captured movements of the arm and hand by recording of reflective markers. These markers were placed on predefined points of the thorax and upper extremity according to the guidelines of the International Society of Biomechanics for the arm^29^ and an adapted version of a validated marker model for the hand (Figure 3).^30^

**Figure 2.**
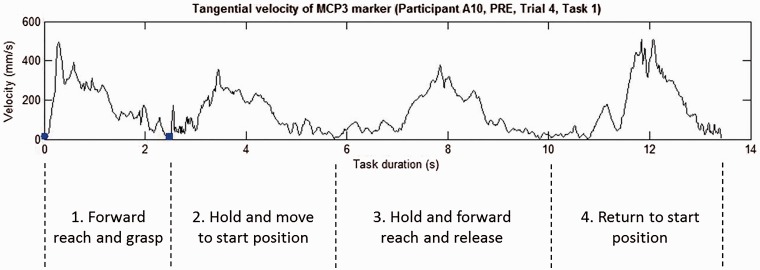
Four phases of the reach and grasp task. Shown is the velocity profile (mm/s) of the hand marker. The blue dot and square represent the start and end of the reach to grasp phase, respectively. MCP3: metacarpophalangeal 3.

The captured VICON data were analyzed using VICON Nexus 1.8.2 and transferred to MATLAB (R2013b, MathWorks Inc., Natick, Massachusetts, USA) for custom, offline analysis. The data were filtered with a second-order low-pass Butterworth filter of 20Hz and zero phase shift. For each participant, the average of the seven repetitions with largest HO was used for further analysis. For participants with fewer useful repetitions available, for example, in case of a poor data sample, the average of at least three useful repetitions was used. For participants without useful data on HO, for example, in case of problems grasping the bottle, the average of the seven middle trials was used in statistical analysis. No data on HO are available for these participants in the results. Data were recorded from all four phases in the reach and grasp task, but only data from the first phase (the reach to grasp phase) were used for analysis (Figure 2). This first phase of the task is most comparable to many activities performed in daily life, and this phase is most relevant for parameters related to the hand. Participants completed the whole task in order to not interfere fluent performance of the reach to grasp task.

**Figure 3.**
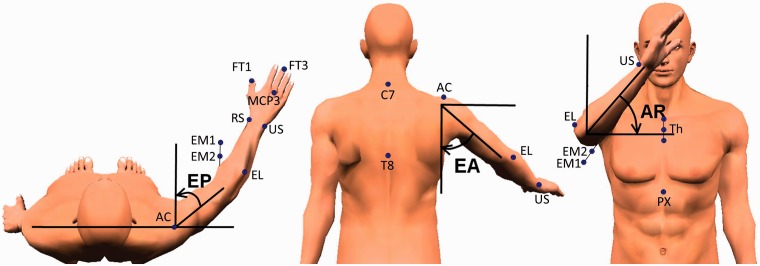
Joint angles of the shoulder and marker positions. Source: adapted from Krabben et al.^33^ EP: elevation plane, EA: elevation angle, AR: axial rotation; PX: processus xiphoideus; C7: 7th cervical vertebra; T8: 8th thoracal vertebra; Th: thorax markers on a triangular frame with Th1 = upper marker on incisura jugularis, Th2 = middle marker on sternum, Th3 = lower marker on sternum; AC: acromioclavicular joint; EL: lateral epicondyle; EM2: medial epicondyle (proximal marker on pointer); EM1: medial epicondyle (distal marker on pointer); US: ulnar styloid; RS: radial styloid; MCP3: metacarpophalangeal 3; FT1: distal phalanx of the thumb; FT3: distal phalanx of the third finger.

The following kinematic variables were calculated from the 3D position data during this first reach to grasp phase. The maximal velocity (mm/s) was defined as the maximal of the tangential velocity profile of the hand marker (metacarpophalangeal 3 in Figure 3). The MT was defined as the time (s) participants needed to perform the first phase of the reach and grasp task. Movement onset and offset were defined as the moment at which the tangential velocity of the hand marker exceeded and dropped below 2% of the maximal velocity, respectively.^31^ MS was defined as the number of movement units (nmu) in the tangential velocity profile of the hand marker, which was searched for local minima and maxima. According to Alt Murphy et al., a difference between a minimum and next maximum value exceeding the amplitude limit of 20 mm/s indicated a velocity peak, if the time between two subsequent peaks was at least 150 ms.^31^ The maximal HO was determined as the maximal Euclidean distance (mm) between the tip of the thumb and the tip of the middle finger. Although the transport and grasp components predominantly occur simultaneously (in parallel) in most healthy people, stroke patients often prefer serial processing of movements.^32^ The moment of maximal HO relative to the moment of maximal hand speed was used as a measure of temporal pattern using the formula^14^

$$\text{Temporal}\;\text{pattern}=\frac{\text{Moment}\;\text{maximal}\;\text{hand}\;\text{opening}}{\text{Moment}\;\text{maximal}\;\text{velocity}}\times 100\%$$

Forward trunk displacement was defined as the difference between the maximal and minimal forward displacement (mm) of the trunk marker (Th2) in the sagittal plane. Thoracohumeral joint angles were calculated according to the recommendations of the International Society of Biomechanics.^29^ Joint excursions of the elbow, wrist, and shoulder were calculated as the difference between maximal and minimal joint angles (degrees) during the first phase of the reach and grasp task. Elbow flexion and extension excursion was defined as the joint angle between the forearm and the humerus. Wrist flexion and extension excursion was calculated by the angle between the vectors joining the wrist and forearm markers and wrist and hand markers. The shoulder joint orientation (Figure 3) was represented by the elevation plane (EP), elevation angle (EA), and axial rotation (AR). The EP was defined as the angle between the humerus and a virtual line through the shoulders, viewed in the transversal plane. The EA represented the angle between the humerus and thorax, in the plane of elevation. The AR was defined as the rotation around a virtual line from the glenohumeral joint to the elbow joint.

### Statistical analyses

Statistical analyses were performed using IBM SPSS Statistics 22 for Windows with level of significance set at α < 0.05. All outcome measures were inspected for normal distribution using histogram plots including normal curves and normal probability plots, and Shapiro–Wilk tests, prior to selection of appropriate statistical tests. Descriptive statistics (mean with standard deviation) were used for all outcome measures.

We consulted a statistician for advice on statistical analysis of relationships. We inspected normal curves before choosing the appropriate test, especially considering the small sample size. Relationships between clinical outcome measures (FM, ARAT, and MAL), grip strength and kinematic variables at pre were evaluated using Pearson's or Spearman's correlation coefficient, based on distribution of the data. To examine which predictor or combination of predictors explained the greatest amount of variance in clinical outcome measures, multiple linear regression with forward deletion was used. Only kinematic variables which showed strong significant correlations (r ≥ 0.70)^34,35^ with the clinical assessments were entered into the regression model, in addition to known kinematic variables which show correlations with clinical outcome measures after stroke.^17,18^ Probability for entry in forward regression was set at 0.05 and removal at 0.10. Prior to these analyses, tests were done to ensure no violation of assumptions of normality, linearity, and homoscedasticity by checking histogram plots including normal curves and normal probability plots of the residuals and the scatterplot of standardized residuals against standardized predicted values. Multicollinearity among the predictors was checked by inspecting the individual correlations among predictors and tested by the criterion of a variance inflation factor greater than 10. In cases of very strong correlations (≥0.80)^34^ between predictors, one predictor is substituted for another.

Since training-related outcomes are described elsewhere,^22^ no statistics on training-induced changes between sessions and between groups are performed and reported here. However, correlation analyses were performed for training-induced changes on clinical outcome measures (FM, ARAT, and MAL) and changes in grip strength and kinematic variables using Pearson's or Spearman's correlation coefficient.

## Results

### Participants

Cross-sectional baseline data from 20 participants were analyzed. From 15 of those participants, post-training data were available as well, divided randomly between a control group (N = 8) and experimental group (N = 7). Participants showed a large variation of stroke severity, ranging from severely to mildly impaired patients, based on baseline FM score.^36^ Considering participant characteristics at baseline (Table 1), there were no differences between the two training groups.

**Table 1.** Participant characteristics at baseline (absolute numbers or mean (standard deviation)).

	Cross-sectional measurement PRE	Training data PRE (N = 15)	
	All participants N = 20	Control N = 8	Experimental N = 7
Gender	12 male/8 female	3 male/5 female	6 male/1 female
Age in years	60 (11)	61 (10)	57 (12)
Months post-stroke	21 (18)	27 (26)	16 (10)
Type of stroke	16 infarction/ 3 hemorrhage/ 1 unknown	6 infarction/ 2 hemorrhage	7 infarction/ 0 hemorrhage
Affected body side	12 left/8 right	4 left/4 right	5 left/2 right
Dominant arm	3 left/17 right	2 left/6 right	1 left/6 right
Fugl-Meyer score (maximal 66 points)	40 (15)	38 (12)	40 (18)
Action Research Arm Test score (maximal 57 points)	28 (18)	26 (16)	33 (21)
Stroke severity	7 mild/8 moderate/ 5 severe	2 mild/4 moderate/ 2 severe	2 mild/3 moderate/ 2 severe

### Cross-sectional measurement pre training

#### Correlations

**Figure 4.**
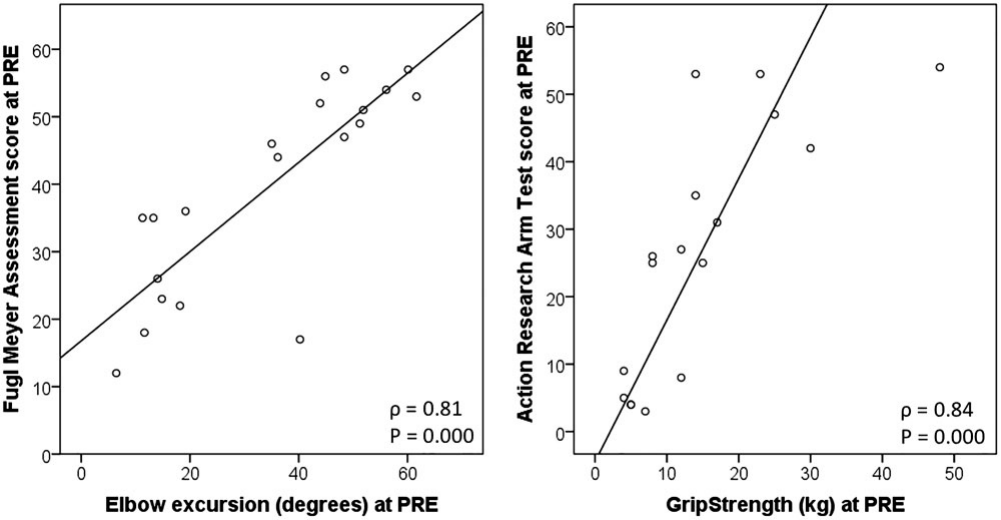
Scatterplots of Fugl-Meyer score with elbow excursion (left) and Action Research Arm Test with Grip strength (right).

Sensorimotor function (FM) correlated strongly (r ≥ 0.70) with grip strength, maximal velocity, and elbow excursion. FM correlated moderately (r ≥ 0.40) with MT (negatively), maximal HO, trunk displacement (negatively), and shoulder excursion AR (Table 2). The same kinematic variables correlated with activity capacity (ARAT), with comparably strong associations (Table 2). Self-perceived activity performance (MAL) correlated with grip strength, maximal HO, and elbow excursion and shoulder excursion AR, but the associations were slightly weaker for elbow excursion (Table 2). Examples of relations between a clinical outcome measure (FM, ARAT) and kinematic variable (elbow excursion, grip strength) are displayed in Figure 4.

**Table 2.** Correlation coefficients between clinical outcome measures and kinematic variables.

	FM score (max 66)^a^	ARAT score (max 57)	MAL AOU (0–5)	MAL QOM (0–5)
Grip strength (kg)^a^	**0.85 (N = 17)**	**0.84 (N = 17)**	**0.70 (N = 17)**	**0.82 (N = 17)**
Movement time (s)^a^	**−0.50 (N = 20)**	**−0.51 (N = 20)**	−0.34 (N = 17)	−0.30 (N = 17)
Smoothness (nmu)^a^	−0.16 (N = 20)	−0.21 (N = 20)	−0.06 (N = 17)	−0.02 (N = 17)
Maximal hand opening (mm)	**0.51 (N = 16)**	**0.54 (N = 16)**^a^	**0.57 (N = 13)**	**0.56 (N = 13)**
Maximal velocity (mm/s)	**0.75 (N = 20)**	**0.63 (N = 20)**	0.30 (N = 17)	0.41 (N = 17)
Temporal pattern (%)	0.07 (N = 16)	0.19 (N = 16)^a^	−0.07 (N = 13)	−0.13 (N = 13)
Trunk displacement (mm)	**−0.65 (N = 20)**	**−0.57 (N = 20)**	−0.38 (N = 17)	−0.39 (N = 17)
Elbow excursion (degrees)	**0.81 (N = 20)**	**0.80 (N = 20)**^a^	**0.56 (N = 17)**	**0.68 (N = 17)**
Wrist excursion (degrees)	0.23 (N = 20)	0.09 (N = 20)	0.09 (N = 17)	0.12 (N = 17)
Shoulder excursion—elevation plane (degrees)	0.26 (N = 20)	0.32 (N = 20)	0.45 (N = 17)	0.48 (N = 17)
Shoulder excursion—elevation angle (degrees)	−0.15 (N = 20)	−0.16 (N = 20)	0.09 (N = 17)	0.10 (N = 17)
Shoulder excursion—axial rotation (degrees)	**0.57 (N = 20)**	**0.64 (N = 20)**	**0.58 N = 17)**	**0.62 (N = 17)**

Note: Significant correlations in boldface.

FM: Fugl-Meyer assessment; ARAT: Action Research Arm Test; MAL AOU: Motor Activity Log Amount of Use; MAL QOM: Motor Activity Log Quality of Movement; nmu: number of movement units.

a Spearman's rho (otherwise Pearson Correlation).

#### Multiple linear regression analyses

Forward multiple regression revealed that one kinematic variable, elbow excursion, explained the largest amount of variance in the assessment of sensorimotor function, explaining 60.3% of the total variance in FM score. Elbow excursion and grip strength together explained 68.4% of the total variance in the assessment of activity capacity (ARAT), with a unique contribution of 12.0% (P = 0.027) and 10.2% (P = 0.039), respectively. In the models of MAL AOU and MAL QOM, grip strength was the only predictor that explained variance in self-perceived activity performance for both models, explaining 33.9% and 40.5% of the variance, respectively (Table 3).

**Table 3.** Results of multiple linear regression analyses (based on N = 17).

Model	Dependent variable	Extracted predictors in forward regression	Unstandardized coefficients		Adjusted R^2^	Model P value
			B	Standard error		
1	FM	Constant	17.209	4.472	0.603	0.000
		Elbow excursion	0.620	0.123		
2	ARAT	Constant	1.682	5.773	0.597	0.000
		Elbow excursion	0.790	0.159		
3		Constant	0.695	5.124	0.684	0.000
		Elbow excursion	0.482	0.195		
		Grip strength	0.723	0.318		
4	MAL AOU	Constant	0.833	0.376	0.339	0.008
		Grip strength	0.062	0.020		
5	MAL QOM	Constant	0.618	0.330	0.405	0.004
		Grip strength	0.062	0.018		

FM: Fugl-Meyer assessment; ARAT: Action Research Arm Test; MAL AOU: Motor Activity Log Amount of Use; MAL QOM: Motor Activity Log Quality of Movement.

### Correlations training-induced changes

In contrast with the correlations found at the pre evaluation measurement, only a few significant correlations were observed in both groups when examining associations between training-induced changes in clinical and kinematic outcome measures on individual level via correlation analysis (Table 4). For the control group, improvements in activity capacity (ARAT) were strongly associated with improvements in maximal HO (ρ = 0.89, P = 0.041). For the experimental group, changes in activity capacity (ARAT) were associated with increased grip strength (ρ = 0.94, P = 0.001). Remarkably, a change in self-perceived activity performance (MAL QOM) was associated negatively with changes in grip strength (ρ = −0.77, P = 0.044), and MAL AOU was associated negatively with changes in wrist excursion (r = −0.94, P = 0.002) and changes in increased shoulder excursion EA (r = −0.91, P = 0.004).

**Table 4.** Correlation coefficients training induced changes clinical outcomes measures and kinematic outcomes for the control group (left) and experimental group (right).

	Control group (N = 8)				Experimental group (N = 7)			
	FM score (max 66)	ARAT score (max 57)^a^	MAL AOU (0–5)	MAL QOM (0–5)	FM score (max 66)	ARAT score (max 57)	MAL AOU (0–5)	MAL QOM (0–5)
Grip strength (kg)	−0.42	0.66	0.33	−0.37	0.75	**0.94**	0.45	**−0.77**
MT (s)^a^	0.05	−0.57	−0.29	0.02	0.30	0.32	−0.28	−0.32
MS (nmu)	0.47	−0.20	0.22	0.56	0.07	0.07	0.10	0.10
Maximal HO (mm)^b^	0.14	**0.89**	0.42	0.59	−0.79	−0.75	0.23	0.80
Maximal velocity (mm/s)	0.02	0.68	0.43	0.25	0.61	0.27	−0.27	−0.40
Temporal pattern (%)^b^	−0.17	0.11	−0.35	−0.09	−0.39	−0.11	0.26	0.30
TD (mm)^a^	0.11	−0.43	0.24	−0.14	−0.08	0.00	−0.52	−0.26
EE (degrees)	−0.03	0.15	0.32	0.12	−0.57	−0.59	0.41	0.72
WE (degrees)	0.25	0.16	0.11	0.10	0.31	0.11	**−0.94**	−0.55
EP (degrees)	0.01	0.15	−0.39	−0.09	0.42	0.40	0.56	−0.13
EA (degrees)	−0.25	0.09	−0.21	−0.10	0.42	0.26	**−0.91**	−0.61
AR (degrees)	−0.22	0.11	0.10	0.09	0.66	0.54	0.29	−0.31

Note: Significant correlations in boldface.

FM: Fugl-Meyer assessment; ARAT: Action Research Arm Test; MAL AOU: Motor Activity Log Amount of Use; MAL QOM: Motor Activity Log Quality of Movement; MT: movement time; MS: movement smoothness; nmu: number of movement units; HO: hand opening; TD: forward trunk displacement; EE: elbow flexion and extension excursion; WE: wrist flexion and extension excursion; EP: elevation plane; EA: elevation angle; AR: axial rotation.

a Spearman's rho (otherwise Pearson Correlation).

b Correlations based on N = 5.

## Discussion

The current study investigated the relationships between an extensive set of objective movement execution kinematics, obtained from 20 chronic stroke patients during a functional reach and grasp task, grip strength and clinical outcome measures on sensorimotor function, activity capacity, and self-perceived activity performance. This evaluation was repeated in 15 stroke patients after six weeks of arm and hand training at home, aimed at obtaining a more in-depth insight into the role of recovery versus compensation in technology-supported training. Almost all movement execution parameters showed strong or moderate relationships with sensorimotor function and activity capacity at the pre measurement, with strongest correlations for grip strength and elbow excursion. The strong relationships seen during the pre measurement were not transferable to relationships considering training-induced changes on individual level.

### Cross-sectional measurement pre training

Previous studies demonstrated high associations among clinical outcome measures such as FM and ARAT.^37–39^ Associations of kinematic outcomes with clinical outcome measures have also been examined^17–19^ but with a limited set of movement execution parameters. In the current study, strong relationships were found for movement execution parameters and grip strength with sensorimotor function and activity capacity. The relationships with self-perceived activity performance in daily life were weaker, which is comparable to previous research.^17^ In the current study, elbow excursion was the most significant contributor to the variance in FM score (60.3%), and a combination of elbow excursion and grip strength explained the majority of variance (68.4%) in ARAT score.

Other studies indicated that MS and total MT, together with compensatory trunk displacement, associated best with ARAT^17^ and trunk displacement with FM.^18^ However, outcomes involving hand grip strength^17,18^ and elbow excursion (range of motion)^17^ were not included in these above-mentioned studies. We also found moderate correlations for FM and ARAT with MT and trunk displacement, but elbow excursion and grip strength showed stronger associations in the regression models when all of these parameters were considered. It can be argued that compensatory trunk displacement might be reflected in decreased elbow excursion. Indeed, initial inspection of the data for multicollinearity showed a moderate negative correlation between trunk displacement and elbow excursion, which supports these findings in the context of previous research.

The present findings on pre correlations and corresponding determinants strongly suggest that it is valuable for treatment applications in neurorehabilitation after stroke to consider targeting at least elbow excursion and hand grip strength, i.e. both reaching and grasping. This is in agreement with studies indicating that task-specific, functional exercises have a high potential to stimulate functional improvements.^40^

### Correlations training-induced changes

It is recognized that individual data display large variations between stroke patients, also in terms of amount of change after a (technology-supported) intervention.^41^ Therefore, correlation analysis of change scores, considering individual cases, might reveal a more realistic picture in understanding which factors may contribute to functional improvements and in which patients. In this regard, the findings in the current sample suggest that improvements in activity capacity after training were most associated with improvements in hand function (either in terms of range of motion or strength). However, the absolute differences were small. This makes it difficult to fully address the aim of whether mechanisms of recovery and/or compensation were involved.

Surprisingly, the self-perceived performance measure showed different results than the capacity measure. A change in MAL after training was negatively correlated with changes in grip strength, wrist excursion, and shoulder excursion EA in the experimental group, which is difficult to interpret in context of the current study. This is possibly because of the subjective nature of the MAL or the MAL measuring other constructs than actual performance. Potentially, participants could relate this to other qualities of movement. Further, improvements on function, capacity, and self-perceived performance might not occur simultaneously.^42^

In general, chronic stroke patients are thought to benefit most from task-specific interventions,^43^ involving both reaching and grasping, which was the approach for both training interventions.^22^ The fact that changes in activity capacity were associated with predominantly hand function improvements, whereas no substantial hand function improvements were evident on group level after technology-supported training, suggests that this intervention apparently did not sufficiently target hand function. Although the intervention with the current sample did not provide the desired information, we expect that the current analysis approach is useful to give more insight into the underlying mechanisms of recovery and compensation after stroke. In addition, it provides more specific directions for design of (technology-supported) interventions for arm and hand function. We recommend targeted interventions, particularly addressing functional movements involving both reaching (elbow excursion) and hand function (grip strength or HO) after stroke. Moreover, evaluation measurements should be included that address these particular endpoints. This is in line with expert consensus gained for guidelines facilitating standardized assessment of such interventions, which proposed inclusion of technologies as assessment tools besides clinical scales.^10^ This consensus approach highlighted the need for more information about useful data derived from such technological methods, for which the current findings provide a first indication.

### Limitations

A limitation of our study concerns the relatively small sample size (N = 20) for the correlations and multiple linear regression analyses pre-training and for the data on correlations of training-induced changes (N = 15). Elbow excursion and grip strength together were the most important predictors in the model of ARAT. For multiple linear regression models, a minimum of 10 observations per predictor variable will generally allow good estimates.^44^ Since the model of ARAT was based on only 17 participants, there is a possibility of overfitting of the model, which should be taken into account when interpreting the results. A higher number of participants will increase the power of the study. The risk with our number of participants might result in a slight overestimation of the effect size and slightly decrease in reproducibility of the results. However, our results give a first indication of tailored treatment applications to stimulate functional improvements after stroke.

Further, the current training intervention was performed with chronic stroke patients at home, with somewhat more impaired participants than previous studies measuring correlations between kinematic and clinical outcomes,^17^ which limits the generalizability of the results to other patient groups in other settings. In practice, these interventions should be considered at an earlier stage after stroke, where larger treatment effects would be expected in the subacute phase.^45^

## Conclusion

Moderate to strong relationships of movement execution parameters and grip strength with FM and ARAT were found, with strongest contributions for grip strength and elbow excursion. The findings imply that the inclusion of both reaching (elbow excursion) with hand function (grip strength or HO) might be valuable targets for (technology-supported) treatment applications to stimulate functional improvements after stroke. To which extent this was successful in the current technology-supported intervention remains unclear due to limited training-induced changes, but it does provide directions for design of (technology-supported) interventions for arm and hand function. We recommend targeted interventions addressing functional movements involving both arm and hand movements simultaneously and including objective measures of elbow excursion and hand function (grip strength and HO) to evaluate their effects.
